# Rapid and Efficient Functionalized Ionic Liquid-Catalyzed Aldol Condensation Reactions Associated with Microwave Irradiation

**DOI:** 10.3390/ijms15011284

**Published:** 2014-01-17

**Authors:** Chang Wang, Jing Liu, Wenguang Leng, Yanan Gao

**Affiliations:** Dalian Institute of Chemical Physics, Chinese Academy of Sciences, Dalian 116023, China; E-Mails: chill@dicp.ac.cn (C.W.); liujing207@dicp.ac.cn (J.L.); lengwg@dicp.ac.cn (W.L.)

**Keywords:** ionic liquid, green chemistry, aldol reaction, microwave

## Abstract

Five quaternary ammonium ionic liquid (IL) and two tetrabutylphosphonium ILs were prepared and characterized. An environmentally benign and convenient functionalized ionic liquid catalytic system was thus explored in the aldol condensation reactions of aromatic aldehydes with acetone. The aldol reactions proceeded more efficiently through microwave-assisted heating than through conventional thermal heating. The yield of products obtained under microwave heating for 30 min was approximately 90%, and the ILs can be recovered and reused at least five times without apparent loss of activity. In addition, this catalytic system can be successfully extended to the Henry reactions.

## 1. Introduction

Green chemistry, that possesses the spirit of sustainable development, has been booming since 1991 and continues to attract more and more interest [1]. The utilization of organic solvents in chemical processes, many of which are volatile, flammable, and toxic, is irreconcilable with the aim of environmental protection and sustainability. Thus, the search for green and efficient reaction protocols is still imperative. Ionic liquids (ILs) are the most explored source of alternative solvents. The great interest in such compounds is due to the fact that they have attractive properties such as negligible vapor pressure, chemical and thermal stability, non-flammability, high conductivity and the ability to act as catalysts [2,3]. More importantly, they are “designable solvents” since functional groups can be incorporated to impart them particular properties or reactivities [4]. The term “task-specific ionic liquids” (TSILs) has been introduced to describe such ILs and they have been applied to various organic reactions as environmentally preferable solvents, reagents, or catalysts [5–9].

Aldol reactions are an effective means of forming C–C bonds and the products of α,β-unsaturated ketones are frequently found in complex polyol architectures of natural products. These reactions are highly atom-economic and have been studied extensively [10–14]. Generally, the synthetic approach to aldol reaction involves the use of organic solvents and acidic or basic catalysts. However, the presence of strong acid or base makes this condensation process suffer from reverse and side reactions, thus giving low yields to the corresponding products [15,16]. For this reason, improved methodologies have been developed that use various Lewis acids [17], Lewis or Brønsted bases [18–20], heterogeneous catalysts [21] and complexes of metal ion [22–25], each affording variable yields of aldol condensation products. However, one or more of the disadvantages such as the use of hazardous agents and organic solvents necessitate the development of greener and more efficient protocols.

ILs have been reported to be useful aldol reaction media [26–45]. For example, the self-aldol condensation of propanal to form 2-methylpent-2-enal in a series of imidazolium based ILs has been intensively studied by Mehnert *et al.* [28], where NaOH was used as strong base and increased yields of oligomers > C_9_ were found compared with molecular solvents. A direct method to unsymmetrically substitute bis(arylidene)alkanones by sequential, selective condensation reactions in a distillable IL, namely dimethylammonium dimethylcarbamate (DIMCARB), was reported by Rosamilia *et al.* [29]. Han and co-workers have systematically investigated the direct aldol reactions in a series of guanidine-based TSILs [31,32]; the effect of anions on the aldol reactions was detected and the highest catalytic activity was obtained in acetate based IL among 9 TSILs [32]. Subsequently, they synthesized a new IL, (2-hydroxyethyl)-trimethyl-ammonium (*S*)-2-pyrrolidinecarboxylic acid ([Choline][Pro]) from biorenewable and nontoxic raw materials, which has shown to be an efficient catalyst for the direct aldol reactions [33]. Moreover, using proline, piperdine or prolinamide derivatives as basic catalysts gave a high conversion of aldol condensation whilst maintaining a good selectivity in several imidazolium based ILs [34–45]. The improved catalytic performance in the ILs was considered to be due to the stabilization of the iminium intermediate formed from the ketone and the secondary amine of the catalyst or because of the enhanced nucleophilicity of the enamine [34]. Therefore, a series of functionalized ILs incorporated with pyrrolidine and proline units have been synthesized and tested as organocatalysts for direct aldol reactions [46–49]. These new TSILs were found to effectively catalyze the direct aldol reactions of cyclic ketone or acetone and various aromatic aldehydes. In addition, our recent work has demonstrated that the cross-aldol condensation reactions of aromatic aldehydes with cyclopentanone proceeded efficiently in several amine-functionalized ILs [50].

Even so, limitations such as long reaction times, use of toxic organic compounds (TOCs) as additives, complicated post-treatment process and difficulty of separating condensation product from reaction system still need to be solved.

**Figure 1.**
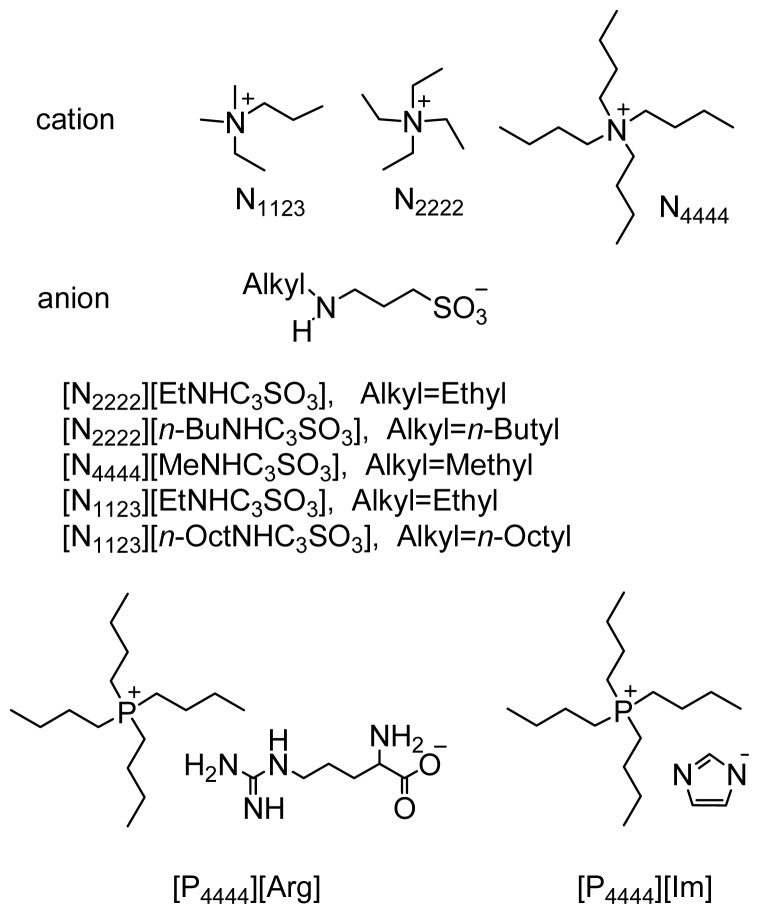
Structures of functionalized ILs.

Microwave-assisted organic synthesis has received increasing attention as a valuable alternative to the conventional heating to speed up chemical reactions [51–58]. It provides fast heating rates and enables rapid optimization of the reaction procedures. Herein, a green and efficient functionalized IL catalytic system was developed to facilitate the aldol condensation reactions through the use of microwave heating technology. The structures of functionalized ionic liquids are shown in Figure 1. The aldol reactions proceeded quickly and generally offered good to excellent yields and also can be extended successfully to the Henry reactions. The short reaction times and simple reaction conditions coupled with a broad substrate scope render the protocol particularly attractive for the efficient preparation of bioactive and medicinally interesting molecules.

## 2. Results and Discussion

### 2.1. Effect of Solvents on the Aldol Condensation Reaction

**Scheme 1.**
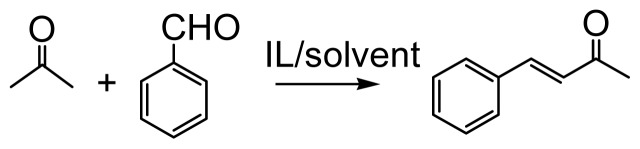
Model aldol reaction of benzaldehyde and acetone.

We describe here the aldol condensation reactions between various aromatic aldehydes and acetone in the functionalized ILs. Our first investigation focused on the model reaction of benzaldehyde and acetone in the amine-functionalized IL [N_2222_][EtNHC_3_SO_3_] (Scheme 1). The desired 4-phenylbut-3-en-2-one (**3a**) was isolated in a yield of 28% when only [N_2222_][EtNHC_3_SO_3_] was used as solvent/catalyst; a possible reason for this is the high viscosity of the IL, which slows down the mass transfer in the catalytic system. Hence, we began our investigation by the evaluation of solvents that would decrease the viscosity of the reaction system. We attempted to add an additional 50 wt % of acetone to decrease the viscosity, and also avoid the effect of additives. A 74% conversion was observed, suggesting that viscosity may play an important role in the aldol reaction. We then added 50 wt % of ethanol instead of acetone, however, only 5% conversion of benzaldehyde was observed. This indicates the importance of solvents in the catalytic process. In contrast, when 50 wt % of H_2_O was added to the model reaction system, aldol product was obtained in a conversion of more than 99% with high selectivity. It thus can be seen that water is an ideal solvent for the aldol reactions. Hence, all aldol condensation reactions in this work were carried out in a solution of 50 wt % IL and 50 wt % water, unless specified otherwise. The conversion and yield of the model reaction in the presence of different solvents are summarized in Table 1. Our previous work also indicated that water in the aldol condensation reactions can promote the enolate intermediate catalytic cycle, thereby accelerating the reaction rate and suppressing the by-product reaction pathway. In addition, an improved yield of aldol reactions achieved through the addition of water was also reported by others [51–58].

### 2.2. Comparison of the Aldol Condensation Reactions under Different Heating Conditions

**Figure 2.**
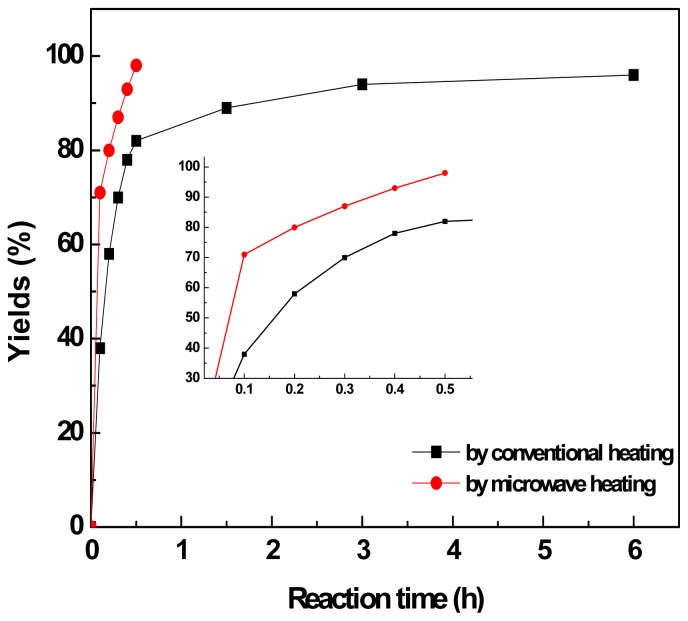
Comparison of the yields of product **4a** under the conditions of microwave heating and conventional heating. Reaction conditions: 2.5 mmol benzaldehyde, 7.5 mmol acetone, 3.4 mmol [N_2222_][EtNHC_3_SO_3_] (1.0 g) and 50 wt % of H_2_O (1.0 mL H_2_O), 80 °C.

We then compared the aldol reactions under different heating conditions. Figure 2 shows the kinetic profiles in the aldol condensation of model reaction. For clarification, the kinetic profiles in 30 min are shown in the insert in Figure 2. We can see that the complete conversion was almost finished in 30 min when using microwave-assisted heating. However, it spent about 6 h to reach the final 95% conversion when using traditional oil-bath heating under the same temperature condition. Obviously, the reaction proceeded more rapidly to give product **3a** by microwave-assisted heating than conventional oil-bath heating, suggesting that the microwave worked more efficiently than conventional heating method. For microwave synthesis, it is known that the reaction medium must have an adequately high dielectric constant (ɛ) for microwave absorption. Alternatively, ILs are ideal candidates for making the nonpolar solvent suitable for microwave heating [59]. The ionic character of ILs provides excellent coupling capability with microwave irradiation. It has been reported that ILs could be used as a microwave-absorbing assistant in organic synthesis, including aldol condensation reactions [58,60]. It is worthy noticing that, in addition to the model reaction, almost all aldol reactions in this work gave much faster conversion by microwave-assisted heating than conventional oil-bath method. Similar to the model reaction, the other aldol reactions in this work can be basically finished in 30 min by microwave-assisted heating, whereas the time is generally more than 3 h if conventional thermal heating is used instead. These results showed that microwave-assisted heating is more efficient for the IL-catalyzed aldol reactions.

### 2.3. Comparison of the Aldol Condensation Reactions in Different ILs

In view of the excellent yields observed in the amine-functionalized [N_2222_][EtNHC_3_SO_3_] catalytic system described here, the model reactions in other ILs involving amine-functionalized [N_2222_][*n*-BuNHC_3_SO_3_], [N_4444_][MeNHC_3_SO_3_], [N_1123_][EtNHC_3_SO_3_], [N_1123_][*n*-OctNHC_3_SO_3_] and two basic tetrabutylphosphonium ILs [P_4444_][Im] and [P_4444_][Arg] were also carried out for comparison (Table 2). The model reaction gave results of both high conversion and selectivity in different IL catalytic systems and the reaction proceeded with not less than 90% conversions to benzaldehyde and around 90% yields, except the [N_1123_][*n*-OctNHC_3_SO_3_] catalytic system. The yields obtained in the [N_2222_][EtNHC_3_SO_3_], [N_4444_][MeNHC_3_SO_3_], [P_4444_][Im], and [P_4444_][Arg] systems are relatively higher than those afforded in the [N_1123_][EtNHC_3_SO_3_] and [N_1123_][*n*-OctNHC_3_SO_3_] systems. The results reveal that IL has an obvious effect on the aldol condensation reactions. It is well known that the physicochemical properties of ILs can be adjusted by choosing different cation/anion structure and therefore, many ILs have been designed for a specific purpose for reactions [61]. Although [N_1123_][EtNHC_3_SO_3_] has the same cation with [N_1123_][*n*-OctNHC_3_SO_3_], the conversion for benzaldehyde in [N_1123_][EtNHC_3_SO_3_] is 90%, much higher than that in [N_1123_][*n*-OctNHC_3_SO_3_], where a 68% conversion was observed. This suggests that the anion of IL with octyl chain does not favor the aldol condensation reactions. The reason could be the steric hindrance of the long alkyl chain. Moreover, we cannot exclude the possibility that [*n*-OctNHC_3_SO_3_] has a relatively weaker basicity than [EtNHC_3_SO_3_].

### 2.4. Recycling of the Functionalized ILs

**Figure 3.**
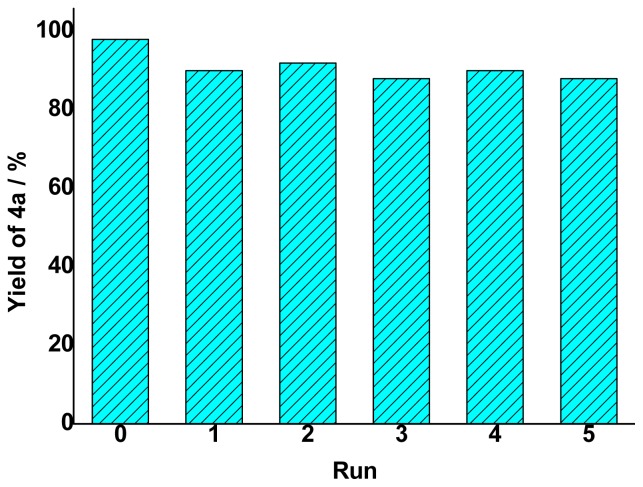
Recycling of [N_2222_][EtNHC_3_SO_3_] aqueous solution in the model aldol reaction. Reaction conditions: 2.5 mmol benzaldehyde, 7.5 mmol acetone, 3.4 mmol (1.0 g) [N_2222_][EtNHC_3_SO_3_] and 50 wt % of H_2_O (1.0 mL), 80 °C, 0.5 h.

After extraction of the products by chloroform three times, the remaining water and IL were collected for the next cycle without further treatment. Investigations into the model reaction of benzaldehyde and acetone showed that the recovered IL could be successfully reused at least five times without obvious loss of activity (Figure 3). The yields of product **3a** were maintained about 90% in the next five cycles, which is almost as high as their first use. It is worth noting that a very small amount of unreacted reactants are present in the reused aqueous IL solution in every recycle because the reactants are soluble in the IL-water medium. In fact, it is not necessary to remove the reactants thoroughly in every step because the activity of the ILs was not affected by the residues and the unreacted substrates can proceed to the next cycle. Importantly, all functionalized ILs studied here can be reused for the aldol reactions without obvious loss of reactivity.

### 2.5. Influence of Different Substituents on Aromatic Aldehydes

**Scheme 2.**
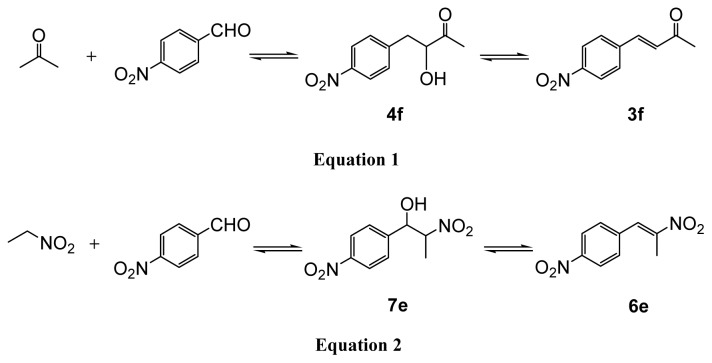
The reaction processes of aldol and Henry reaction when 4-nitrobenzaldehyde was used as reactant.

Aldol reactions of aromatic aldehydes with different substituents and acetone catalyzed by [N_2222_][EtNHC_3_SO_3_] were also studied. To detect the effect of substituents on aromatic aldehydes on the reaction, *p*-tolualdehyde and *p*-methoxybenzaldehyde (bearing electron-donating methyl and methoxyl groups) and 2-chrolobenzaldehyde and 3-bromobenzaldehyde (containing electron-withdrawing chloride and bromide groups) were chosen as the substrates. The results are listed in Table 3. It can be seen that the benzaldehyde, 2-chrolobenzaldehyde and 3-bromobenzaldehyde can undergo an aldol condensation process effectively, giving the corresponding product α,β-unsaturated ketones in excellent yields (close to 100%). This is because introducing the electron-withdrawing groups onto aromatic aldehydes favors the nucleophilic addition of a ketone enolate to the aldehydes to form the aldol products, which has been discussed in detail in our previous work [50]. As expected, the reactions with less reactive *p*-tolualdehyde and *p*-methoxybenzaldehyde provided a relatively lower yield of 92% and 88% respectively. However, we found that the conversion is low (<5%) and no α,β-unsaturated ketone product was obtained when 4-nitrobenzaldehyde was used as reactant. The reason is that 4-nitrobenzaldehyde is not soluble in the solution of 50 wt % [N_2222_][EtNHC_3_SO_3_] and 50 wt % water. To this end, we used the same amount of ethanol instead of water to get a homogeneous reaction solution. The result showed that the conversion for 4-nitrobenzaldehyde reached 98% under the same conditions. Interestingly, although the conversion is high, the selectivity is much different. In the case of IL/water system, the yields of α,β-unsaturated ketone product were good regardless of which type of ILs and aldehydes are used. The aldol reactions in the functionalized ILs conform to the base-catalyzed mechanism [47] and the resultant β-hydroxy ketones usually dehydrate to give the unsaturated carbonyl compounds [Scheme 2, Equation (1)] Nevertheless, in the case of [N_2222_][EtNHC_3_SO_3_]/ethanol system, the selectivity of β-hydroxy ketone product [4-(4-nitrophenyl)-4-hydroxy-2-butanone, **4f**] is 91%, much higher than that of 8% for α,β-unsaturated ketone product **3f**. When using conventional thermal heating, a similar result was obtained but with a long heating time of 6 h. We also investigated the aldol condensation of butanone and 4-nitrobenzaldehyde. The yield of β-hydroxy ketone product is 95%, a similar result to that of acetone reaction system; the reason is not known at present.

### 2.6. Extension to the Henry Reactions

Additionally, we proceeded to examine the utility of functionalized ILs in the Henry reaction, also referred to as the nitro-aldol reaction. Henry reactions of aromatic aldehydes with different substituents and nitroethane catalyzed by [P_4444_][Im] with 50 wt % water as solvent were studied. The results are listed in Table 4. Microwave heating works more efficiently than a conventional thermal heating method, similarly to results previously noted. The Henry reactions were finished in 30 min by microwave-assisted irradiation but more than 3 h were spent in oil-bath heating under the same temperature condition. In this section, the molar ratio of nitroethane to aromatic aldehydes is 1:1, that is, nitroethane is not excessive. Even so, the conversion of all aromatic aldehydes is almost 100% with a high yield of more than 95% except for 4-nitrobenzaldehyde where although the yield is high, there is no β-nitro alcohol product obtained. In contrast, the conversion of 4-nitrobenzaldehyde is 88% and the yield of β-nitro alcohol (l-(4-nitrophenyl)-2-nitropropan-l-ol, **7e**) and nitroalkene (**6e**) product is 76% and 12%, respectively, when ethanol was used instead of water [Scheme 2, Equation (2)]. This result is similar to that of the aldol reaction of 4-nitrobenzaldehyde, where the β-hydroxy ketone product is much higher than that of the enone product, as stated above. It should be noted that we also used a 50 wt % ethanol to increase the solubility of reactants in this system. Considering that water would generally suppress the dehydration, it is reasonable to believe that nitro group on the aromatic aldehyde may lead to a high selectivity for β-hydroxy ketone inthe aldol reaction and β-nitro alcohol in the Henry reaction.

## 3. Experimental Section

### 3.1. General Information

Reagents and solvents were obtained from commercial suppliers and were used without further purification. All the functionalized ILs and reaction products were characterized using NMR spectroscopy. The aldol and Henry products were characterized by ^1^H NMR and ^13^C NMR spectra were recorded with a Bruker Advance III 400 MHz NMR spectrometer (Bruker BioSpin Corporation, Fällanden, Switzerland) at 298 K, using the solvent residual peak as internal standard. All microwave irradiation reactions were carried out on a Microwave SYNTH Plus manufactured by Milestone company (Sorisole, Italy), operating with continuous irradiation power of 400 W.

### 3.2. Synthesis and Characterization of Functionalized ILs

The synthesis of all ILs used in this work has been previously reported (Figure 1) [61–64]. Particularly, all amine-functionalized quaternary ammonium ILs were synthesized according to our previously reported methods [50]. Tetrabutylphosphonium ILs, tetrabutylphosphonium l-arginine ([P_4444_][Arg]) and tetrabutylphosphonium imidazol ([P_4444_][Im]), were synthesized as described by Ohno and Dai, respectively [61,63].

[N_2222_][EtNHC_3_SO_3_]: ^1^H NMR (400 MHz, D_2_O, δ, ppm): 3.26 (q, *J* = 7.3 Hz, 8H), 2.91 (t, *J*_1_ *=* 8.8 Hz, *J*_2_ *=* 6.9 Hz, 2H), 2.66 (t, *J =* 7.4 Hz, 2H), 2.59 (q, *J =* 7.2 Hz, 2H), 1.94–1.85 (m, 2H), 1.29–1.23 (m, 12H), 1.06 (t, *J* = 7.2 Hz, 3H); ^13^C NMR (100 MHz, D_2_O, δ, ppm): 51.56, 48.79, 46.58, 42.42, 23.62, 13.14, 6.23.

[N_2222_][*n*-BuNHC_3_SO_3_]: ^1^H NMR (400 MHz, D_2_O, δ, ppm): 3.29 (q, *J* = 7.3 Hz, 8H), 3.01–2.89 (t, 2H), 2.71 (t, *J* = 7.4 Hz, 2H), 2.60 (t, *J =* 7.3 Hz, 2H), 2.00–1.87 (m, 2H), 1.55–1.43 (m, 2H), 1.37 (m, *J*_1_ *=* 14.7 Hz, *J_2_* = 7.3 Hz, 2H), 1.29 (t, *J =* 7.1 Hz, 12H), 0.93 (t, *J =* 7.3 Hz, 3H); ^13^C NMR (100 MHz, D_2_O, δ, ppm): 51.92, 49.13, 48.11, 47.20, 30.65, 23.90, 19.84, 13.26, 6.56.

[N_4444_][MeNHC_3_SO_3_]: ^1^H NMR (400 MHz, D_2_O, δ, ppm): 3.20 (m, 8H), 2.93 (m, 2H), 2.69 (t, *J =* 7.3 Hz, 2H), 2.35 (s, 3H), 1.92 (m, *J*_1_ *=* 9.9 Hz, *J*_2_ *=* 7.5 Hz, 2H), 1.65 (m, *J*_1_ *=* 15.8 Hz, *J*_2_ *=* 7.9 Hz, 8H), 1.36 (m, 8H), 0.95 (t, *J =* 7.4 Hz, 12H); ^13^C NMR (100 MHz, D_2_O, δ, ppm): 57.79, 48.61, 33.91, 23.21, 22.81, 18.84, 12.54.

[N_1123_][EtNHC_3_SO_3_]: ^1^H NMR (400 MHz, D_2_O, δ, ppm): 3.39 (q, *J =* 7.3 Hz, 2H), 3.31–3.18 (m, 2H), 3.05 (s, 6H), 2.99–2.89 (m, 2H), 2.70 (t, *J =* 7.4 Hz, 2H), 2.62 (q, *J =* 7.2 Hz, 2H), 1.94 (m, *J*_1_ *=* 15.3 Hz, *J*_2_ *=* 7.7 Hz, 2H), 1.86–1.71 (m, 2H), 1.36 (t, *J =* 7.3 Hz, 3H), 1.09 (t, *J* = 7.2 Hz, 3H), 1.00 (t, *J =* 7.3 Hz, 3H); ^13^C NMR (100 MHz, D_2_O, δ, ppm): 64.90, 59.58, 49.82, 49.18, 46.96, 42.76, 24.03, 15.55, 13.56, 9.88, 7.43.

[N_1123_][*n*-OctNHC_3_SO_3_]: ^1^H NMR (400 MHz, D_2_O, δ, ppm): 3.34 (q, *J =* 7.3 Hz, 2H), 3.25–3.14 (m, 2H), 3.00 (s, 6H), 2.87 (m, *J*_1_ *=* 9.1 Hz, *J*_2_ *=* 6.8 Hz, 2H), 2.69–2.59 (t, 2H), 2.59–2.49 (t, 2H), 1.89 (m, *J*_1_ *=* 15.3 Hz, *J*_2_ *=* 7.7 Hz, 2H), 1.81–1.67 (m, 2H), 1.57–1.40 (m, 2H), 1.36–1.18 (m, 13H), 0.95 (t, *J* = 7.3 Hz, 3H), 0.86 (t, *J* = 6.6 Hz, 3H); ^13^C NMR (100 MHz, D_2_O, δ, ppm): 64.82, 59.54, 49.84, 49.19, 49.01, 47.61, 31.65, 29.21, 29.11, 28.77, 27.11, 24.18, 22.44, 15.52, 13.74, 9.85, 7.41.

[P_4444_][Arg]: ^1^H NMR (400 MHz, DMSO, δ, ppm): 2.89 (m, *J* = 7.0 Hz, 3H), 2.79 (m, 1H), 2.17 (m, *J* = 12.3 Hz, 8H), 1.43 (m, 20H), 0.93 (t, *J* = 6.8 Hz, 12H); ^13^C NMR (100 MHz, D_2_O, δ, ppm): 179.71, 158.60, 56.34, 41.59, 34.18, 27.90, 27.26, 26.92, 24.15, 24.02, 23.88, 23.85, 23.73, 23.18, 23.14, 18.11, 17.63, 13.98, 13.70.

[P_4444_][Im]: ^1^H NMR (400 MHz, DMSO, δ, ppm): 7.01 (s, 1H), 6.61 (s, 2H), 2.25–2.11 (m, 1H), 1.33–1.55 (m, 16H), 0.92 (t, *J* = 7.1 Hz, 12H); ^13^C NMR (100 MHz, D_2_O, δ, ppm): 141.52, 124.38, 23.91, 23.75, 23.17, 23.15, 18.05, 17.58, 13.73.

### 3.3. General Procedure for the Aldol and Henry Reactions

**Scheme 3.**
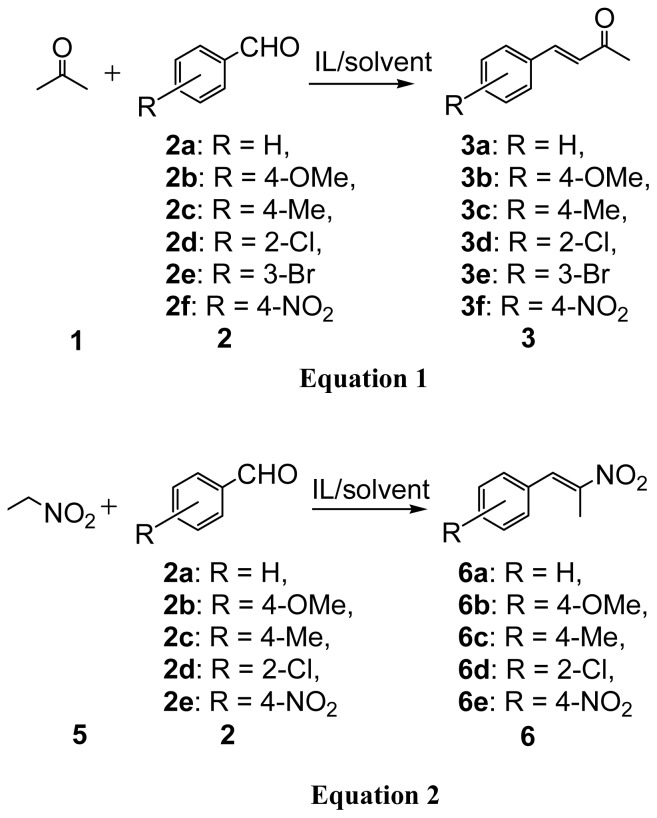
Aldol and Henry reactions.

A mixture of aromatic aldehydes (2.5 mmol) and excess acetone (7.5 mmol) was added to a solution of functionalized IL (3.4 mmol) [Scheme 3, Equation (1)] with a certain amount of water (or ethanol). The resulting mixture was stirred and heated in a 20 mL Q20 quartz microwave vial sealed with a Teflon^®^ crimp top (Milestone company, Sorisole, Italy). As comparison, the same reactions were also carried out by a conventional oil-bath heating. At completion, the reaction mixture was extracted with chloroform (3 × 10.0 mL) and the IL-rich phase was left to reuse for next cycle reactions. The organic layers were combined and washed with water to remove any remaining IL and dried over anhydrous MgSO_4_. The solvent was removed under vacuum and the residue was purified by flash column chromatography on silica gel using ethyl acetate/hexanes as the eluent. Conversions based on aromatic aldehydes and yields were calculated by ^1^H NMR spectroscopic measurements. The same reaction processes, post-treatment and characterization were explored in the Henry reaction by use of aromatic aldehydes (2.5 mmol) and nitroethane (2.5 mmol) [Scheme 3, Equation (2)].

All aldol products are known compounds [33,65–67].

4-Phenylbut-3-en-2-one (**3a**): ^1^H NMR (400 MHz, CDCl_3_, δ, ppm): 7.54 (m, 2H), 7.50 (d, 1H), 7.39 (m, 3H), 6.70 (d, *J* = 16.3 Hz, 1H), 2.37 (s, 3H).

4-(4-Methoxyphenyl)but-3-en-2-one (**3b**): ^1^H NMR (400 MHz, CDCl_3_, δ, ppm): 7.54–7.49 (m, 3H), 6.95 (d, *J* = 8.4 Hz, 2H), 6.64 (d, *J* = 16.2 Hz, 1H), 3.87 (s, 3H), 2.39 (s, 3H).

4-(4-Methylphenyl)but-3-en-2-one (**3c**): ^1^H NMR (400 MHz, CDCl_3_, δ, ppm): 7.49 (d, *J* = 16.3 Hz, 1H), 7.44 (d, *J* = 8.0 Hz, 2H), 7.20 (d, *J* = 8.0 Hz, 2H), 6.98 (d, *J* = 16.2 Hz, 1H), 2.38 (s, 3H), 2.37 (s, 3H).

4-(2-Chlorophenyl)but-3-en-2-one (**3d**): ^1^H NMR (400 MHz, CDCl_3_, δ, ppm): 7.93 (d, *J* = 16.4 Hz, 1H), 7.63 (q, *J* = 1.6 Hz, 1H), 7.43 (q, *J* = 0.8 Hz, 1H), 7.34–7.26 (m, 2H), 6.67 (d, *J* = 16.4 Hz, 1H), 2.42 (s, 3H).

4-(3-Bromophenyl)but-3-en-2-one (**3e**): ^1^H NMR (400 MHz, CDCl_3_, δ, ppm): 7.68 (s, 1H) 7.52–7.41 (m, 3H), 7.27 (q, *J* = 3.2 Hz, 1H), 6.70 (d, *J* = 16.3 Hz, 1H), 2.38 (s, 3H).

4-(4-Nitrophenyl)-3-buten-2-one (**3f**): ^1^H NMR (400 MHz, CDCl_3_, δ, ppm): 8.25 (d, *J* = 8.6 Hz, 2H), 7.69 (d, *J* = 8.6 Hz, 2H), 7.53 (d, *J* = 16.2 Hz, 1H), 6.81 (d, *J* = 16.2 Hz, 1H), 2.42 (s, 3H).

4-(4-Nitrophenyl)-4-hydroxy-2-butanone (**4f**): ^1^H NMR (400 MHz, CDCl_3_, δ, ppm): 8.15 (d, *J* = 8.8 Hz, 2H), 7.51(d, *J* = 8.8 Hz, 2H), 5.23 (m, 1H), 3.71 (d, *J* = 3.2 Hz, 1H), 2.84 (d, *J* = 6.4 Hz, 2H), 2.20 (s, 3H).

All Henry products are also known compounds [68–70].

1-Phenyl-2-nitropropene (**6a**): ^1^H NMR (400 MHz, CDCl_3_, δ, ppm): 8.12 (s, 1H), 7.35–7.28 (m, 5H), 2.49 (s, 3H).

1-(4-Methoxyphenyl)-2-nitropropene (**6b**): ^1^H NMR (400 MHz, CDCl_3_, δ, ppm): 8.05 (s, 1H), 7.43 (d, *J =* 8.0 Hz, 2H), 6.98 (d, *J =* 9.0 Hz, 2H), 3.87 (s, 3H), 2.48 (s, 3H).

1-(4-Methylphenyl)-2-nitropropene (**6c**): ^1^H NMR (400 MHz, CDCl_3_, δ, ppm): 8.11 (s, 1H), 7.38 (d, *J =* 8.0 Hz, 2H), 7.29 (d, *J =* 8.0 Hz, 2H), 2.49 (s, 3H), 2.44 (s, 3H).

1-(2-Chlorophenyl)-2-nitropropene (**6d**): ^1^H NMR (400 MHz, CDCl_3_, δ, ppm): 8.21 (s, 1H), 7.51 (dd, *J_1_* = 2.0 Hz, *J_2_* = 7.0 Hz, 1H), 7.41–7.29 (m, 3H), 2.37 (s, 3H).

2-Nitro-1-(4-nitro)phenylpropene (**6e**): ^1^H NMR (400 MHz, CDCl_3_, δ, ppm): 8.31 (d, *J =* 8.5 Hz, 2H), 7.59 (d, *J =* 8.5 Hz, 2H), 8.09 (br, s, 1H), 2.46 (d, *J* = 1.0 Hz, 3H).

l-(4-Nitrophenyl)-2-nitropropan-l-ol (**7e**): ^1^H NMR (400 MHz, CDCl_3_, δ, ppm): 8.22 (d, *J* = 10.0 Hz, 2H), 7.48 (d, 2H), 5.21 (d, *J* = 10.0 Hz, 2H), 4.81 (m, lH), 3.5 (s, 1H), 1.42 (d, *J* = 10.0 Hz, 3H), 1.27 (d, *J* = 10.5 Hz, 3H).

## 4. Conclusions

In summary, we have developed an efficient and improved strategy for aldol reactions in functionalized ionic liquid (IL) catalytic systems by using microwave-assisted heating. In comparison to the conventional thermal heating, microwave heating was found to work more efficiently. The aldol condensation reactions generally offered good to excellent yields and also can be extended successfully to the Henry reactions. In addition, our methodology offers several substantial advantages, such as, no toxic chemicals were used in the reaction process and the utilization of the functionalized ILs as both solvents and catalysts does not require any strong acids/bases to catalyze the aldol reactions. The functionalized ILs catalyzed the aldol reactions for a broad range of substrates with high yields and can be reused at least five times without apparent loss of activity.

## Figures and Tables

**Table 1.** Aldol reactions of benzaldehyde and acetone (model reaction) in different catalytic systems containing 50 wt % [N_2222_][EtNHC_3_SO_3_] and 50 wt % solvent. Reaction conditions: 2.5 mmol benzaldehyde, 7.5 mmol acetone, 1.0 g [N_2222_][EtNHC_3_SO_3_], microwave temperature 80 °C, 0.5 h.

Solvent	Conversion (%)	Yield (%)
None	28	13
Ethanol	5	44
Acetone	74	46
Water	>99	98

**Table 2.** Model aldol reactions in different functionalized IL catalytic systems. Reaction conditions: 3.4 mmol ILs, 50 wt % of H_2_O of catalytic systems, 2.5 mmol benzaldehyde, 7.5 mmol acetone, 80 °C, 0.5 h.

Entry	IL	Conversion (%)	Yield (%)
1	[N_2222_][EtNHC_3_SO_3_]	>99	98
2	[N_2222_][*n*-BuNHC_3_SO_3_]	95	85
3	[N_4444_][MeNHC_3_SO_3_]	>99	98
4	[N_1123_][EtNHC_3_SO_3_]	90	80
5	[N_1123_][*n*-OctNHC_3_SO_3_]	68	64
6	[P_4444_][Im]	96	88
7	[P_4444_][Arg]	97	87

**Table 3.** Aldol reactions of various aromatic aldehydes with acetone in the [N_2222_][EtNHC_3_SO_3_] catalytic systems. Reaction conditions: 3.4 mmol ILs (1.0 g), 50 wt % of H_2_O (1.0 mL) of catalytic system, 2.5 mmol aromatic aldehydes, 7.5 mmol acetone, 80 °C, 0.5 h.

Entry	R	Product	Conversion (%)	Yield (%)
1	H	**3a**	>99	98
2	4-OMe	**3b**	88	87
3	4-Me	**3c**	92	89
4	2-Cl	**3d**	>99	96
5	3-Br	**3e**	>99	91
6	4-NO_2_	**3f**	<5	0
7	4-NO_2_^a^	**3f**	98	8

a 1.0 g ethanol was used instead of 1.0 g H_2_O.

**Table 4.** Henry reactions of various aromatic aldehydes with acetone in the [P_4444_][Im] catalytic systems. Reaction conditions: 3.4 mmol ILs, 50 wt % of H_2_O (1.0 mL), 2.5 mmol aromatic aldehydes, 7.5 mmol acetone, 80 °C, 0.5 h.

Entry	R	Product	Conversion (%)	Yield (%)
1	H	**6a**	>99	98
2	4-OMe	**6b**	>99	87
3	4-Me	**6c**	>99	89
4	2-Cl	**6d**	>99	96
5	4-NO^2^	**6e**	<5	0
6	4-NO_2_^a^	**6e**	88	12

a 5.0 mL ethanol was used instead of 1.0 mL H_2_O.
